# Surface Immobilization of TiO_2_ Nanotubes with Bone Morphogenetic Protein-2 Synergistically Enhances Initial Preosteoblast Adhesion and Osseointegration

**DOI:** 10.1155/2019/5697250

**Published:** 2019-03-26

**Authors:** Ying Li, Yunjia Song, Aobo Ma, Changyi Li

**Affiliations:** Stomatological Hospital, Tianjin Medical University, Tianjin 300070, China

## Abstract

Although titanium (Ti) alloys have been widely used as implant materials, the bioinertness of pristine Ti impairs their bioactivity and early osseointegration. In the present work, we prepared TiO_2_ nanotubes (TNT) layer on the titanium (Ti) surface by anodic oxidation. The anodized surface was functionalized with human bone morphogenetic protein-2 coating to form the hBMP-2/TNT surface. The release behavior of hBMP-2 on the hBMP-2/TNT surface displayed a controlled and sustained pattern, compared to that on the hBMP-2/Ti surface, which showed a rapid release.* In vitro* cellular activity tests demonstrated that both TNT and hBMP-2/Ti surfaces, particularly the hBMP-2/TNT surface, enhanced adhesion, proliferation, and differentiation of osteoblast cells. Increased cell adhesion, improved cytoskeleton organization, and immunofluorescence staining of vinculin were observed on the modified surfaces. The TNT, hBMP-2/Ti, and hBMP-2/TNT surfaces, especially the hBMP-2/TNT surface, further displayed an upregulated gene expression of adhesion and osteogenic markers* vinculin*, collagen type 1, osteopontin, and osteocalcin, compared to the pristine Ti surface.* In vivo* experiments using a rat model demonstrated that the TNT and hBMP-2/Ti surfaces, in particular the hBMP-2/TNT surface, improved osseointegration and showed a superior bone bonding ability compared to Ti. Our study revealed a synergistic role played by TiO_2_ nanotubes nanotopography and hBMP-2 in promoting initial osteoblast adhesion, proliferation, differentiation, and osseointegration, thus suggesting a promising method for better modifying the implant surface.

## 1. Introduction

Titanium (Ti) and its alloys are commonly used as dental and orthopedic implant materials because of their good biocompatibility, appropriate mechanical properties, and corrosion resistance [1, 2]. The early osseointegration and initial stability of Ti-based dental implants play an essential role in implant success, which has remarkable and practical clinical significance [3]. Moreover, initial cell adhesion to biomaterials serves as the first step in tissue-implant interactions and is of fundamental importance in the subsequent biological processes such as cell proliferation, differentiation, bone formation, and ultimate osseointegration [4]. Since the bioinertness of Ti hinders its cell/material interaction and early osseointegration [5], development of modified surfaces to improve the biocompatibility of Ti implant materials is of great value.

Recently, many efforts have been applied to alter the surface topography, hydrophilicity, and biological properties of Ti implant surfaces [6–8]. Among these techniques, anodic oxidation (AO) method has attracted much attention. AO is a simple, economical, and versatile technique to produce nanoscale structure and gain improved hydrophilicity on a Ti-based substrate [9, 10]. The ordered and controllable TiO_2_ nanotube surface produced by anodization has been reported to effectively promote osteoblast cell adhesion, proliferation, and differentiation* in vitro* [11–14]. Several studies also indicated that TiO_2_ nanotube layer could enhance osseointegration* in vivo *[15, 16].

Since the natural bone is composed of inorganic and organic components with nanoscale structures, endowing the TiO_2_ nanotube surface with additional chemical or biological features to mimic the architecture of natural bone becomes a promising way to improve cell response and osseointegration. We previously studied the cellular response and bone binding ability of the combination of TiO_2_ nanotube surface with chemical component hydroxyapatite (HA). It was revealed that the nanotopography and HA coating played a synergistic role in promoting osteoblast adhesion, proliferation, differentiation, and osseointegration [17].

In the present study, we further explore the biological effects of the TiO_2_ nanotube surface immobilized with biomolecules. The TiO_2_ nanotubes structures formed onto the Ti surface could be regarded as an ideal reservoir for drug delivery [18]. Moreover, the established TiO_2_ nanotube arrays can be functionalized through combining with biomolecules to acquire synergistic effects [19]. Among these biomolecules, recombinant human bone morphogenetic protein-2 (hBMP-2), which plays a vital role in bone development and regeneration, has been widely used for its good osteoinductivity [20]. BMP-2 not only regulates the osteogenic differentiation of MSCs, but also stimulates osteoprogenitor cells to differentiate into mature osteoblasts [21] and promotes bone formation* in vivo* [22–24].

A controlled release of hBMP-2 from the implant surface is desirable to persistently exert the osteogenic function of hBMP-2 and avoid adverse effects, such as bone overgrowth, ectopic bone formation, and immune responses [25, 26]. Various methods have been developed to acquire a sustained release of BMP-2 from dental and orthopedic implants [27]. However these methods have a few drawbacks, such as the potential risk of host tissue damage cause by organic solvents employment [28] and the difficulty to understand the precise chemical composition and pH-dependent behavior of the dopamine used for immobilization [29]. Therefore, there is an urgent need to develop a simple, safe, and effective method for BMP-2 coating on titanium surface.

Since osteoblasts are anchorage-dependent cells, adhesion of osteoblasts plays a critical role in cell proliferation and function, as well as osseointegration at the implant-bone interface [30]. Thus, the success of osseointegration depends on the stable initial adhesion of osteoblasts onto the implant surfaces, which is a crucial prerequisite to subsequent cell functions such as synthesis of extracellular matrix proteins, formation of mineral deposits, and osseointegration on the substrate surface [18]. Consequently, it is of vital importance to investigate the initial cell adhesion property of the BMP-2 immobilized TiO_2_ nanotube surface.

Although several studies reported the promising results of BMP-2 and nanotubes separately, only a few studies showed the effect of combined use of nanotopography and growth factor on implant surface modification [21, 31, 32]. Among these studies, either the release property of BMP-2 from the modified surface was lacking [21, 32] or the cell response of initial adhesion to the combined surface was not described [31].

Hence, in the present study, we employed TiO_2_ nanotube as the reservoir of hBMP-2 for controlled release to maintain the bioactivity of hBMP-2 and to avoid side effects. The release profile of hBMP-2/TNT surface was evaluated, compared to that of the hBMP-2/Ti substrate. Preosteoblast cell adhesion including adherent cell counting and immunofluorescence staining of actin and vinculin were evaluated. In addition, cell proliferation and differentiation were evaluated through MTT assay and alkaline phosphatase (ALP) activity test. Gene expressions of* vinculin*,* collagen type 1* (*COL*),* osteopontin *(*OPN*), and* osteocalcin* (*OCN*) were analyzed to further explore the underlying molecular mechanisms. Furthermore, a rat model was utilized to examine the* in vivo* effects on early osseointegration.

## 2. Materials and Methods

### 2.1. Specimen Preparation

Commercially available pure titanium plates (10 × 10× 1mm^3^) and cylindrical implant rods (diameter of 2mm, length of 2mm) were used for* in vitro* and* in vivo* experiments, respectively. The AO process was performed under 1 M NaF solution at 10 V, for 1 h at room temperature, with a graphite electrode as the cathode. The anodized plate was named TNT surface, while the polished Ti (Ti) sample was used as the control.

The recombinant human bone morphogenetic protein-2 (hBMP-2) was obtained from Sangon Biotech Company (Shanghai, China). The prepared Ti and TNT plates were loaded by 20 *μ*L of the hBMP-2 solution (50 ng/*μ*L) and were lyophilized with an ALPHA 1-2 LD plus freeze-dryer (Martin Christ, Osterode, Germany) as reported [31]. Through this method, the hBMP-2 coating was deposited on the Ti and TNT surfaces to establish the hBMP-2/Ti and hBMP-2/TNT substrates.

### 2.2. Surface Characterization

Surface morphologies of different substrates were observed using scanning electron microscopy (SEM, Hitachi S-4800, Tokyo, Japan). The surface chemical compositions of different surfaces were examined by X-ray photoelectron spectroscopy (XPS; AXIS Nova, Kratos Analytical, Manchester, UK). The hydrophilicity was assessed from measuring the contact angle of deionized water at room temperature.

### 2.3. In Vitro Release Profile of hBMP-2/Ti and hBMP-2/TNT Surfaces

Specimens were incubated in a phosphate-buffered saline (PBS) solution for 1, 4, 7, 14, and 21 days at 37°C, and the amount of rhBMP-2 was measured using the rhBMP-2-specific Quantikine enzyme-linked immunosorbent assay (ELISA) kit (R&D Systems, MN, USA) according to the manufacturer's instructions. The drug concentration was calculated based on the calibration curve, and the release percentage of rhBMP-2 was calculated by dividing the accumulated released amount by the total rhBMP-2 amount.

### 2.4. Cell Culture

The MC3T3-E1 mouse preosteoblast cells (ATCC, CRL-2593, Rockville, MD) were cultured in *α*-MEM medium (Gibco BRL, Grand Island, NY, USA) supplemented with 10% fetal bovine serum (Hyclone, Logan, UT, USA) and 3% penicillin/streptomycin. Cells were incubated in a humidified atmosphere of 5% CO_2_ at 37°C in an incubator.

### 2.5. Cell Adhesion

Cells (2×10^4^ cells/mL) were incubated with different surfaces for 4 hours, washed to remove the nonadherent cells, fixed, and stained with 4′, 6′-diamidino-2-phenylindole (DAPI, Invitrogen, Thermo Fisher Scientific, MA, USA). Five fields of view were photographed at random using an inverted fluorescence microscope (Olympus IX71, Tokyo, Japan). Three different samples were measured for each group, and the cell numbers were calculated* via* the Image-pro Plus J software (ver. 5.0, Media Cybernetics, Silver Spring, USA).

For immunostaining of actin and vinculin, after 4h cell culture, samples were first incubated with rabbit-anti-mouse vinculin monoclonal antibody and then stained by a goat-anti-rabbit secondary antibody (Invitrogen, Thermo Fisher Scientific, MA, USA). After that, samples were stained with Rhodamine Phalloidin and DAPI (Invitrogen, Thermo Fisher Scientific, MA, USA). At last, samples were examined using a confocal laser scanning microscopy (CLSM, Leica, TCS SP5, Wetzlar, Germany).

### 2.6. Cell Proliferation

The MC3T3-E1 cells (2×10^4^ cells/mL) were seeded on different substrates and cultured for 1, 4 and 7 days. Cell proliferation was investigated using the 3-(4,5-dimethylthiazol-2yl)-2,5-diphenyl tetrazolium bromide (MTT) assay (Sigma, St. Louis, MO, USA). The absorbance of the solution from each well was collected and measured using a spectrophotometer (BioTek, Elx800, Winooski, VT, USA) at 490 nm.

### 2.7. Alkaline Phosphatase (ALP) Activity Assay

The MC3T3-E1 cells (2×10^4^ cells/mL) were incubated with substrates for 7 and 14 days, and cell differentiation ability was measured using an ALP activity kit (Sigma-Aldrich, Saint Louis, Missouri, USA) by reading the absorbance at 405 nm with a spectrophotometer (BioTek, Elx800, Winooski, VT, USA). The ALP activity was normalized to the total protein amount which was determined by BCA protein assay kit (Thermo Scientific, Rockford, IL, USA).

### 2.8. Quantitative Real-Time PCR Analysis

Cells with a concentration of 2×10^4^ cells/mL were cultured for 1, 4 and 7 days. Then, Trizol reagent (Invitrogen Life Technologies, Carlsbad, CA, USA) was used to isolate total RNA. After that, the extracted RNA were reversely transcribed to cDNA using RevertAid First Strand cDNA Synthesis Kit (Thermo Fisher Scientific, MA, USA). Purified gene specific primers for* collagen type 1 *(*COL*),* osteopontin* (*OPN*),* osteocalcin* (*OCN*),* vinculin,* and the housekeeping gene,* glyceraldehyde-3-phosphate dehydrogenase* (*GAPDH*), were obtained from Sangon Biotech Company (Shanghai, China), with the primer sets listed in Table 1. Amplification was conducted as mentioned in a previous article [28].

### 2.9. Animal Experiment

The Animal Ethical and Welfare Committee (AEWC) of Tianjin Medical University approved the ethical aspects of the* in vivo* experiment (No. TMUaMEC 2016006). Cylindrical pure Ti rod implants, 2 mm in diameter and 2 mm in length, were used in the animal experiments. For the details of the preparation of rod implant samples with different surfaces, please refer to* 2.1. Specimen Preparation.* Twelve male Sprague Dawley rats (440–470 g, n=3) were randomized into Ti, TNT, Ti/BMP-2, and TNT/BMP-2 groups, with implant rods separately implanted into the femora. After 4 weeks of implantation, rats were sacrificed and the femora with cylindrical implants were harvested.

### 2.10. Histological Analysis and Push-Out Test

The harvested rat femora were fixed, dehydrated and embedded in Technovit 7200 VLC (Heraeus Kulzer GmbH, Wehrheim, Germany). After that, sections were prepared for hematoxylin and eosin (H&E) staining and observed using microscope (Nikon Ni-E, Tokyo, JAPAN). Push-out test was performed to evaluate the bonding strength at the interface of implant and bone tissue using the electromechanical testing machine (Instron 5544, Canton, MA, USA).

### 2.11. Statistical Analysis

Each experiment repeated three times in this study, and data were analyzed using SPSS 14.0 software (USA). A one-way ANOVA followed by the Student-Newman-Keuls* post hoc* test was used to examine the statistical significance of the difference between samples. Differences with* p*<0.05 were considered to be significant.

## 3. Results

### 3.1. Surface Topography

**Figure 1:**
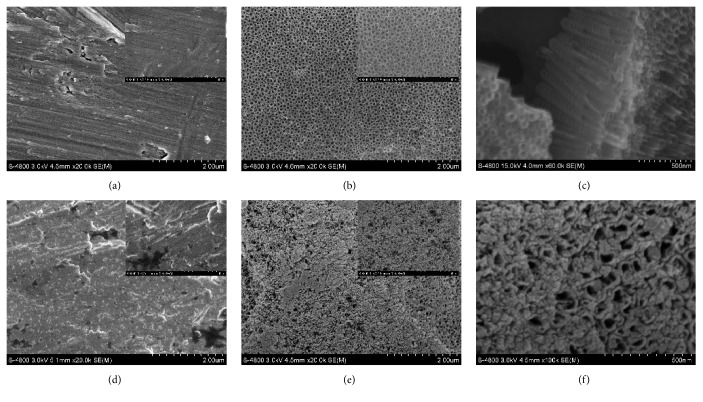
SEM images of Ti (a), TNT (b), hBMP-2/Ti (d), and hBMP-2/TNT (e) samples observed at 20,000 × and 50,000 ×(magnified insets).  Figure 1(c) is the fracture surface of 45 degree tilting sample of the nanotube microstructure; Figure 1(f) is the magnified SEM picture of Figure 1(e)'s inset (100,000 ×).

Figure 1 showed that the pristine Ti displayed a relatively smooth surface with scratch marks caused by the polishing process (Figure 1(a)), while the TNT surface displayed uniform and homogeneous nanotube arrays. The inner diameter of the nanotubes was around 70 nm; the wall thickness was about 5 nm (Figure 1(b)). The fracture surface of 45 degree tilting sample displayed that the depth of the nanotube was about 500 nm in average (Figure 1(b)). The hBMP-2 proteins loosely dispersed on the hBMP-2/Ti surface (Figure 1(d)), whereas the proteins compactly immobilized on the hBMP-2/TNT substrate, covering most of the nanotube openings, thus suggesting higher loading ability for the latter substrate (Figure 1(e)).  Figure 1(f) is the magnified SEM picture of Figure 1(e)'s inset, which further proofed the hBMP-2 adsorption into nanotubes.

### 3.2. Surface Chemical Composition

**Figure 2:**
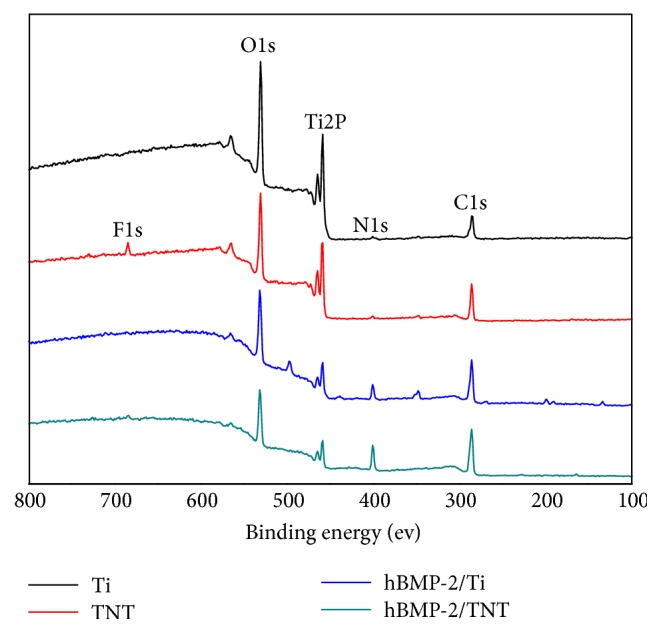
XPS spectra of Ti, TNT, hBMP-2/Ti, and hBMP-2/TNT samples.

Figure 2 and Table 2 showed the surface chemical composition of the different surfaces. The O1s peak is derived from the TiO_2_ layer of the native Ti, while the high content of carbon is partly attributed to the environment contamination. The hBMP-2/Ti (6.08%) and hBMP-2/TNT (4.41%) surfaces display lower percentage of Ti than the pristine Ti (15.61%) and TNT (14.06%) substrates, respectively. Moreover, distinct N1s peaks were shown on both hBMP-2/Ti (9.40%) and hBMP-2/TNT (14.55%) substrates, with higher percentage of N indicating more hBMP-2 immobilization on the latter substrate. Furthermore, the hBMP-2/Ti and hBMP-2/TNT samples showed increased percentages of C element and decreased percentages of O element, as compared to pristine Ti and TNT surfaces, respectively. This can be explained by the incorporation with hBMP-2, as the element content of carbons in attached hBMP-2 is larger than oxygen. These results indicated the successful immobilization of hBMP-2 onto the Ti and TNT surfaces.

### 3.3. Surface Wettability

**Figure 3:**
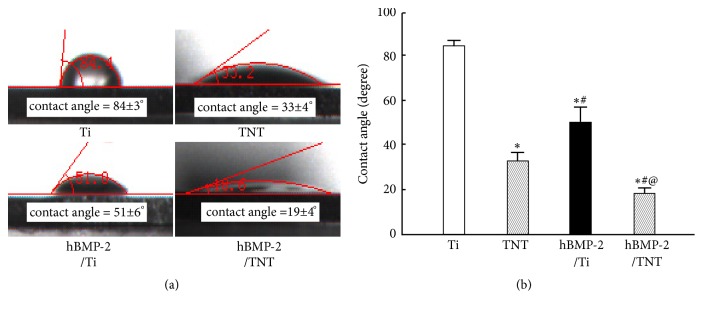
(a) Images of contact angles on Ti, TNT, hBMP-2/Ti, and hBMP-2/TNT samples. (b) Summary of water contact angle degrees on different surfaces. Values are mean ± SD, n=3; *∗*, #, and @ indicate* p* < 0.05 compared with Ti, TNT, and hBMP-2/Ti, respectively.

As shown in Figure 3, the water contact angles of nonanodized surfaces were 84 ± 3° (Ti) and 51 ± 6° (hBMP-2/Ti), respectively, while those of anodized substrates were 33 ± 4° (TNT) and 19 ± 2° (hBMP-2/TNT), respectively. This suggests that AO could remarkably improve the wettability of the originally hydrophobic surfaces. Moreover, decreased contact angles were observed on hBMP-2/Ti (51 ± 6°) and hBMP-2/TNT (19 ± 2°) substrates, as compared to Ti (84 ± 3°) and TNT (33 ± 4°) substrates, mainly attributed to the incorporation of hBMP-2. Interestingly, the lowest contact angle appeared on the hBMP-2/TNT substrate, indicating the synergistic effects of AO and hBMP-2 addition for improving surface hydrophilicity.

### 3.4. Release Behaviors of hBMP-2

**Figure 4:**
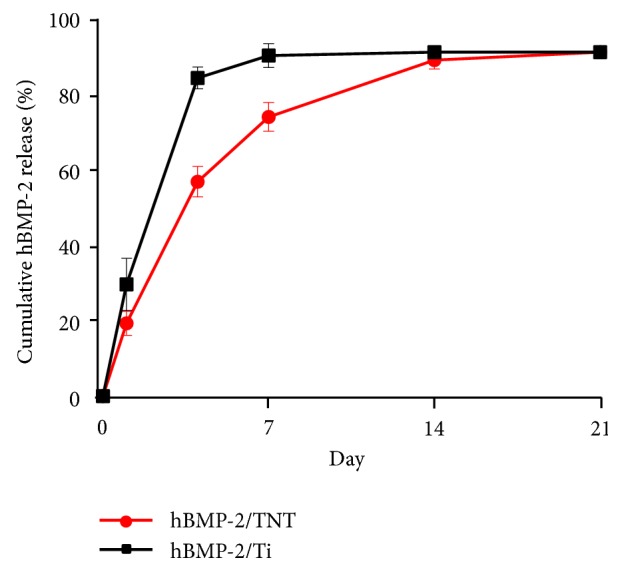
Release behavior of hBMP-2 from hBMP-2/Ti and hBMP-2/TNT surfaces. Samples in PBS (pH 7.4) were monitored for 21 days (values are mean ± SD, n=3).

Figure 4 showed the release behaviors of hBMP-2 from hBMP-2/Ti and hBMP-2/TNT surfaces. The release characteristics of hBMP-2 can be roughly divided into two phases: the initial fast release phase, followed by a slow release phase. In the initial 4 days, the hBMP-2/Ti surface showed a rapid release of 92.5% of hBMP-2. At 7 days, the releasing rate of hBMP-2 reached a plateau, when few hBMP-2 remained in hBMP-2/Ti surface. On the other hand, the hBMP-2/TNT substrate exhibited significantly continuous release profile. As expected, 62.4% was released during the first 4 days, followed by an additional 18.8% release at 7 days, and 16.4% at 14 days, and finally reached a plateau at 21 days. The fast release phase of the hBMP-2/TNT surface is ascribed to the diffusion of the hBMP-2 molecules attached to the top or upper parts of the TiO_2_ nanotubes, while the second phase may be explained by the gradual and controlled diffusion of hBMP-2 molecules from the deeper long nanotube structures [33]. The possible rationale of the substantially improved drug delivery property of nanotube may be due to the more hBMP-2 adsorption into the long tubular structure than the native Ti surface. In addition, the cylindrical shape of nanotube strengthens its resistance to drug release, so as to prolong the drug release period. Moreover, the anodization process during TiO_2_ nanotubes layer fabrication created a very hydrophilic hBMP-2/TNT substrate which may facilitate protein adsorption. Based on the above reasons, the well-organized nanotube structures endowed the hBMP-2/TNT substrate with ability for controlled release of biomolecules such as hBMP-2 for surface functionalization with persistent action.

### 3.5. Cell Adhesion

**Figure 5:**
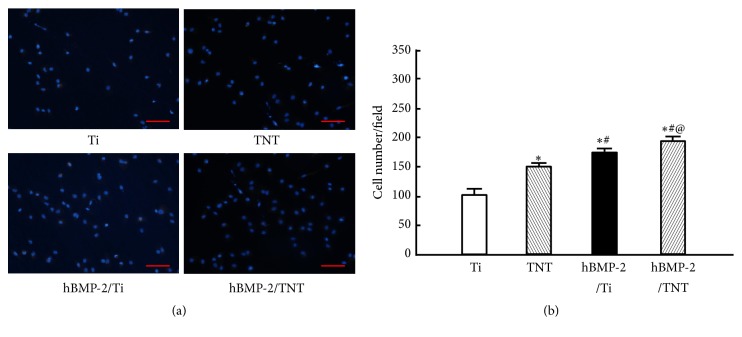
Osteoblast adhesion measured by counting cells stained with DAPI under a fluorescence microscope after 4h of incubation. (a): representative images of Ti, TNT, hBMP-2/Ti, and hBMP-2/TNT samples. (b): summary of adherent cell numbers on different surfaces. Values are mean ± SD, n=3; *∗*, #, and @ indicate* p* < 0.05 compared with Ti, TNT, and hBMP-2/Ti, respectively. Scale bar, 100 *μ*m.

Figure 5 showed that, after 4h incubation, 1.5-fold, 1.7-fold, and 1.9-fold increases of adherent cell numbers were detected on the TNT, hBMP-2/Ti, and hBMP-2/TNT surfaces, respectively, compared to the as-polished Ti (*p*<0.05). The results suggest that both the anodized surface and hBMP-2 coating are able to enhance initial cell adhesion. The highest number of adherent cells was observed on the hBMP-2/TNT surface, which suggests that anodized surface structure and hBMP-2 coating play a synergistic role in promoting the osteoblast adhesion.

**Figure 6:**
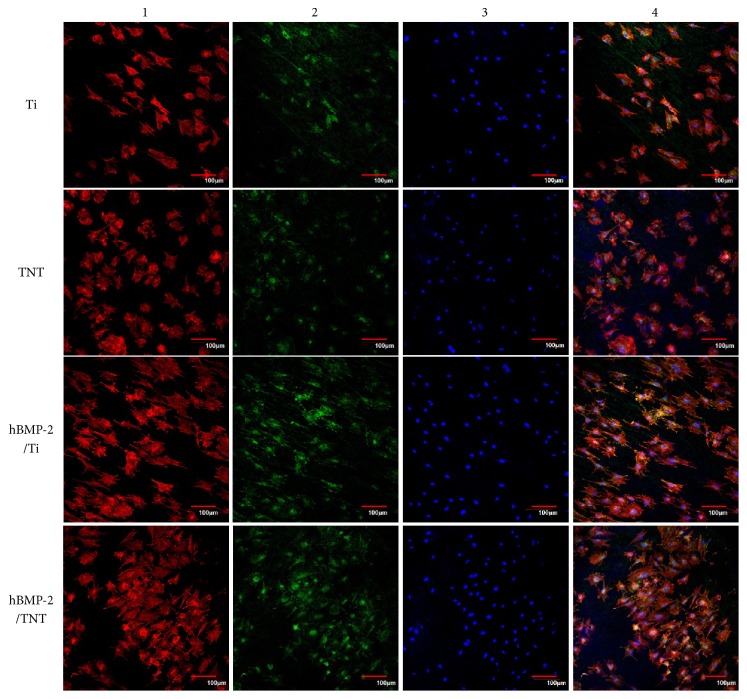
CLSM images of cells cultured on different substrates for 4 h. 1: actin filaments (red), 2: immunofluorescence for vinculin (green), 3: DAPI staining of nucleus (blue), and 4: a merged image of 1, 2, and 3. Scale bar, 100 *μ*m.

The enhanced cell adhesion was further confirmed by immunofluorescence staining of cytoskeleton actin and vinculin, the important marker to identify focal adhesion (FA).  Figure 6 displayed that at 4-hour incubation, cells on the Ti surface tend to elongate parallel to the grinding marks attributed to contact guidance, while on the TNT surface, cells displayed a spread-out cytoskeleton organization, with more stretched actin filaments randomly orientated. Moreover, cells on the hBMP-2/Ti and hBMP-2/TNT surfaces exhibited a further spread-out morphology, compared to the Ti and TNT surface, respectively. Especially on the hBMP-2/TNT surface, cells showed a well-spread cytoskeleton organization, with many actin filaments spreading out with random orientation, which indicates good cell communication. MC3T3-E1 cells grown on the TNT and hBMP-2/Ti surfaces, particularly the hBMP-2/TNT surface, displayed higher protein expressions of vinculin than those cultured on the native Ti. The improved cytoskeletal development and enhanced vinculin protein expression suggested the superior cell adhesion for the hBMP-2/TNT surface due to the combined effects of nanostructure and coating of hBMP-2.

### 3.6. Cell Proliferation and Differentiation

**Figure 7:**
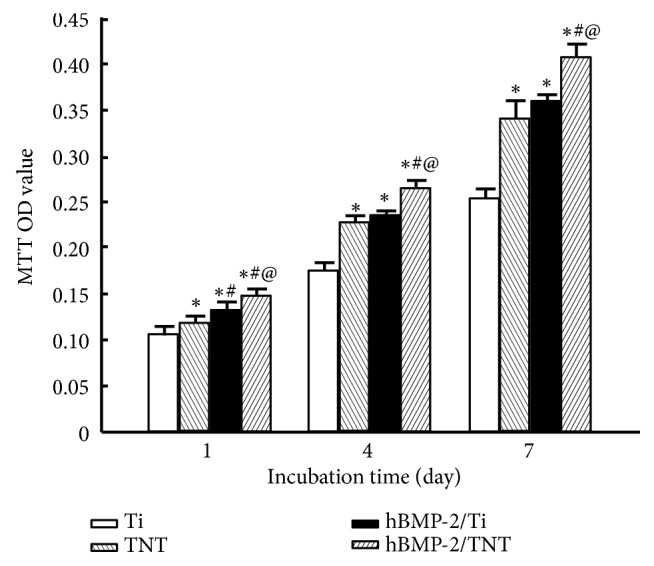
Proliferation of MC3T3-E1 cells on Ti, TNT, hBMP-2/Ti, and hBMP-2/TNT substrates incubated for 1, 4, and 7 days. Values are mean ± SD, n=3; *∗*, #, and @ indicate* p* < 0.05 compared with Ti, TNT, and hBMP-2/Ti, respectively.

Results from the MTT assay revealed that cell numbers displayed a progressive increase for all the specimens over the 7-day period (Figure 7). At each designated time point, cells cultured on TNT, hBMP-2/Ti, and hBMP-2/TNT surfaces exhibited higher proliferation rate compared to that on the pristine Ti,* p*<0.05. Among them, cells grown on the hBMP-2/TNT surface showed the highest proliferation rate, indicating its superior performance in improving cell proliferation.

**Figure 8:**
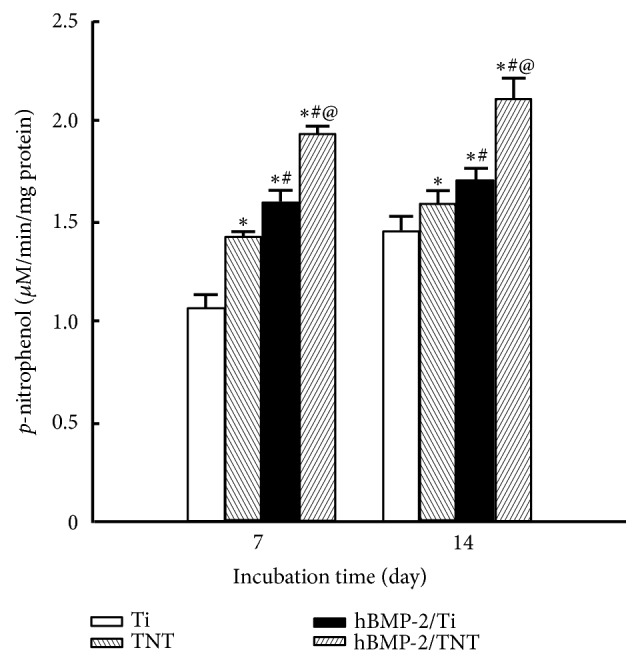
ALP activity of cells on Ti, TNT, hBMP-2/Ti, and hBMP-2/TNT substrates cultured for 7 and 14 days. Values are mean ± SD, n=3; *∗*, #, and @ indicate* p* < 0.05 compared with Ti, TNT, and hBMP-2/Ti, respectively.

As shown in Figure 8, the ALP activity at 14 days was significantly higher than that at 7 days for all of the samples. At a certain time point, the cells on the modified surfaces showed increased ALP activity compared to original Ti surface (*p*<0.05), with cells grown on the hBMP-2/TNT surface which exhibited the highest ALP activity. The results presented above indicate that the ALP activity could be improved by the synergistic function of anodization and growth factor coating of hBMP-2.

### 3.7. Quantitative Real-Time PCR Analysis

**Figure 9:**
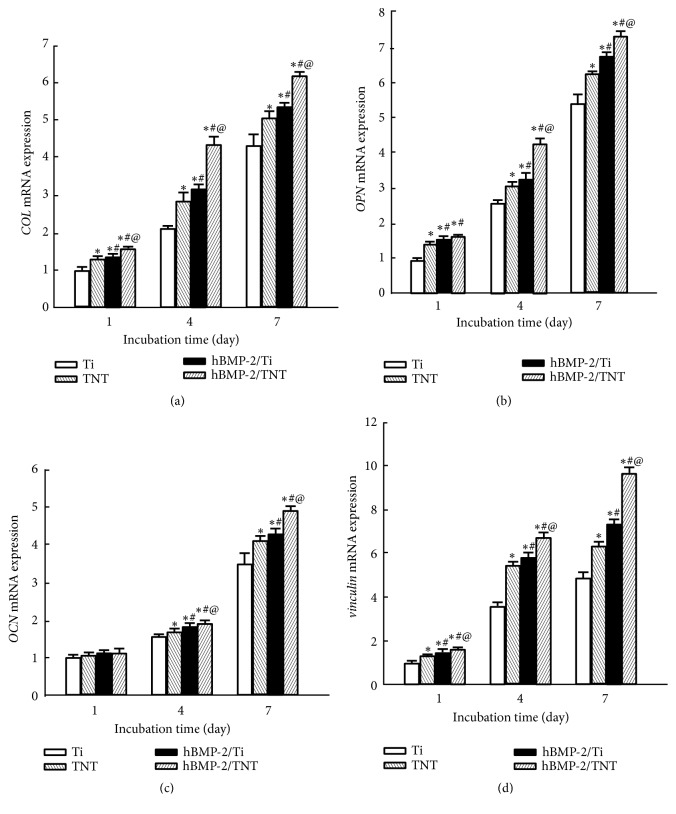
Real-time PCR results representing the MC3T3-E1 cell gene expression levels on the Ti, TNT, hBMP-2/Ti, and hBMP-2/TNT substrates. Values are mean ± SD, n=3; *∗*, #, and @ indicate* p* < 0.05 compared with Ti, TNT, and hBMP-2/Ti, respectively.

As shown in Figure 9, the gene expressions of* COL*,* OPN*,* OCN, and vinculin *in cells on various surfaces increase with incubation time. At each denoted time point, the expression levels of all the interested genes upregulated on the TNT, hBMP-2/Ti, and hBMP-2/TNT surfaces, compared to the Ti surface. In particular, the hBMP-2/TNT surface showed higher gene expression levels than the other surfaces after cultured for 7 days (*p*<0.05). These results demonstrated that the hBMP-2/TNT surface significantly promoted adhesion and osteogenesis-related gene expressions in the osteoblast cells.

### 3.8. In Vivo Experiment

**Figure 10:**
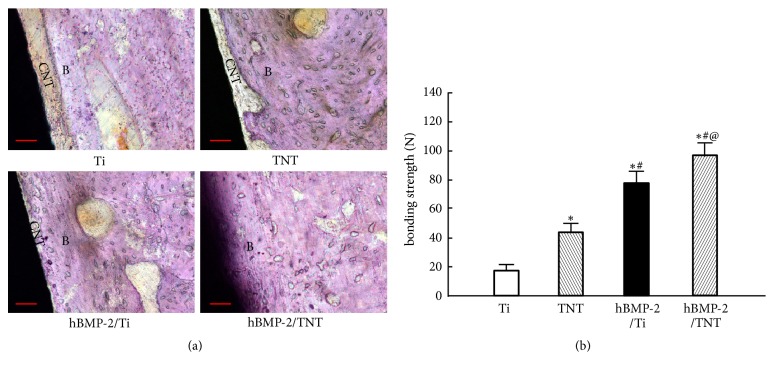
(a) Hematoxylin and eosin (H&E) staining images at the bone-implant interfaces of Ti, TNT, hBMP-2/Ti, and hBMP-2/TNT surfaces after 4-week implantation. CNT: connective tissue; B: bone; scale bar, 200 *μ*m. (b) Push-out tests results. Values are mean ± SD, n=3; *∗*, #, and @ indicate* p* < 0.05 compared with Ti, TNT, and hBMP-2/Ti, respectively.

H&E staining reveals that a relatively thick layer of connective tissues with loose osteoid was formed around the Ti surface, whereas bone trabeculae were developed around the TNT substrate, with a little connective tissue remaining. Moreover, relatively compact bone trabeculae were seen around the hBMP-2/Ti surface, with only scattered connective tissue left. In particular, a large amount of newly generated bones was observed growing energetically at the interface between tissue and the implant with hBMP-2/TNT surface (Figure 10(a)). Furthermore, push-out test indicated that the bonding strengths of TNT, hBMP-2/Ti, and hBMP-2/TNT surfaces were 45 ± 6 N, 78 ± 8 N, and 98 ± 7 N, respectively, compared to that of polished Ti (18 ± 4 N,* p*<0.05, Figure 10(b)). These results indicated excellent osseointegration for the hBMP-2/TNT group.

## 4. Discussion

In this study, the TiO_2_ nanotubes (TNT) layer was functionalized with BMP-2 coating to form the hBMP-2/TNT surface. The modified hBMP-2/TNT surface displayed a sustained release profile of hBMP-2 protein. Our results also revealed that nanostructure and BMP-2 immobilization promote initial cell adhesion, proliferation, differentiation* in vitro,* and osseointegration* in vivo*.

The possible rationale of the substantially improved drug delivery property of TiO_2_ nanotube substrate compared to pristine Ti surface may be explained as follows. First, the formation of a much more hydrophilic hBMP-2/TNT substrate may facilitate protein adsorption. Second, the long tubular structure of TiO_2_ nanotube provides more hBMP-2 storage space than the native Ti surface. Third, the cylindrical shape of nanotube strengthens its resistance to drug release, so as to prolong the drug release period. In view of the above reasons, the hBMP-2/TNT surface fulfilled a more sustained drug release property than the hBMP-2/Ti surface. Future study will be done to further decrease the initial burst release and prolong the drug release period of BMP-2.

It is well-known that the initial cell adhesion, i.e., the first step of implant/tissue interaction, regulates the downstream behavior of cells, such as proliferation and differentiation* in vitro* and in turn affects osseointegration* in vivo *[8, 34]. It is also widely accepted that improved wettability of biomaterials facilitates cell adhesion [35]. Moreover, the addition of hBMP-2 is also shown to facilitate cell spreading [22]. In our study, improved cell attachment, well-formed cytoskeleton structure, and higher expression of FA marker vinculin were observed on the TNT, hBMP-2/Ti, and hBMP-2/TNT substrates, compared to the Ti surface. These phenomena may be explained by the increased surface hydrophilicity on the modified surfaces. The most favorable property of the hBMP-2/TNT substrate for cell adhesion is due to the combined actions of nanostructure and coating of hBMP-2, which generate synergistic effects on optimized initial cell adhesion.

In addition to cell adhesion, the cell proliferation ability investigated by MTT assay showed that cells cultured on the TNT, hBMP-2/Ti and hBMP-2/TNT substrates displayed higher cell proliferation ability than that of the bare Ti surface. These results revealed that the modified topography of nanotube layer and the application of coating with growth factor hBMP-2 could both promote osteoblasts proliferation, similar to previous reports [21, 32]. Furthermore, cell differentiation properties were evaluated by the activity of ALP, a marker of osteoblast early differentiation which promoted cell differentiated into the osteoblasts and is essential to ossification [25, 36]. Similar to previous studies, the TNT and hBMP-2/Ti substrates displayed higher ALP activity than that of bare Ti surface at both 7 and 14 days. Particularly, the hBMP-2/TNT substrate revealed the highest ALP activity among all of the groups. These results mentioned above implied the synergistic effects of nanostructure and hBMP-2 addition in accelerating cell proliferation and differentiation.

We further explored the underlying mechanisms which regulate the improved cell functions of the modified surfaces through measuring the gene expression levels of adhesion and osteogenic markers. Vinculin is a membrane cytoskeletal protein which plays a vital role in activating cell adhesion and cytoskeleton development. It also promotes integrin clustering and composes and stabilizes focal adhesion (FA) [37]. Previous studies suggested that nanotopography formed by AO induces higher cell adhesion in osteoblast cells [14, 38]. hBMP-2 is also shown to be able to induce higher vinculin protein expression in osteoblasts [32]. In accordance with previous reports, our data revealed a continuously increased gene expression of adhesion marker* vinculin* on the TNT, hBMP-2/Ti, and hBMP-2/TNT surfaces during the 7-day observation period. Moreover, the hBMP-2/TNT surface exhibited the highest* vinculin* gene expressions than the other surfaces, at each denoted time point.

Collagen (COL) serves as a marker of osteoblast cell early differentiation and is associated with early osteoid formation [39]. OPN is a well-known marker of middle-stage osteogenic differentiation and participate in later ECM mineralization [40]. OCN is a marker of osteoblast differentiation during the late-stage [41] and signifies the beginning of mineralization [42]. Consistent with previous reports [43], our results elicited the continuous increase of three osteoblast differentiation marker genes (*COL*,* OPN,* and* OCN*) on the TNT, hBMP-2/Ti and hBMP-2/TNT substrates, compared with that on the Ti surface, with the highest expression levels observed on the hBMP-2/TNT surface. These results proved the synergistic effects of nanotopography and hBMP-2 coating to promote adhesion and osteogenesis-related gene expressions.

To further verify the osteogenic potential of modified surfaces, we employed a rat model to investigate the osseointegration properties* in vivo*. It is reported that the anodized surface could not only promote cell attachment so as to improve osseointegration [38], but also inhibit bacterial adhesion, which has adverse effects on the success of implant biomaterials [44]. Furthermore, precious study revealed that 70 nm is the optimum size of TiO_2_ nanotube diameter for osteoconductivity and osseointegration [27]. It has also been reported that BMP-2 has considerable potential not only to stimulate bone formation but also to promote implant osseointegration [45]. In line with those studies, we revealed better bone formation on the TNT and hBMP-2/Ti surfaces. Particularly, we demonstrated superior bone formation ability and enhanced bonding strength at the interface between the hBMP-2/TNT surface and tissue, which further confirmed the* in vitro* results.

## 5. Conclusions

We fabricated a novel hydrophilic hBMP-2/TNT substrate which displayed sustained drug release profile compared to the hBMP-2/Ti surface. The modified hBMP-2/TNT surface facilitates cell adhesion, proliferation, and differentiation in vitro, with continuously enhanced gene expressions of adhesion and differentiation markers. Furthermore, in vivo animal experiment also confirmed the superior osteogenic ability of the modified hBMP-2/TNT surface. Our study suggested that the hBMP-2/TNT surface possess a superior property to improve initial preosteoblast cells adhesion and enhance implant osseointegration, which provides a promising strategy for designing future implants.

## Figures and Tables

**Table 1:** Primers used for real-time PCR analysis.

Gene	Gene bank ID	DNA primer	Sequence	Size (bp)
*GAPDH*	NM_008084.3	Forward	5′-GGTGAAGGTCGGTGTGAACG-3′	233
		Reverse	5′-CTCGCTCCTGGAAGATGGTG-3′	
*COL*	NM_007742.4	Forward	5′-TAAGGGTCCCCAATGGTGAGA-3′	203
		Reverse	5′-GGGTCCCTCGACTCCTACAT-3′	
*OPN*	NM_001204203.1	Forward	5′-CTCACATGAAGAGCGGTGAG-3′	174
		Reverse	5′-TCTCCTGGCTCTCTTTGGAA-3′	
*OCN*	NM_007541.3	Forward	5′-GGACCATCTTTCTGCTCACTCTG-3′	131
		Reverse	5′-GTTCACTACCTTATTGCCCTCCTG-3′	
*vinculin*	NM_009502.4	Forward	5′-GATGCTGGTGAACTCAATGA-3′	171
		Reverse	5′-CGAATGATCTCGTTAATCTC-3′	

Table 1 is reproduced from Li Ying et al. (2019, see [17]).

**Table 2:** Chemical compositions of each sample measured by XPS.

substrates	C%	O%	Ti%	N%	F%
Ti	28.87	54.07	15.61	1.45	0
TNT	38.88	41.99	14.06	1.49	3.59
hBMP-2/Ti	49.74	34.78	6.08	9.40	0
hBMP-2/TNT	55.20	25.12	4.41	14.55	0.72

## Data Availability

The data used to support the findings of this study are available from the corresponding author upon request.
